# Extracellular Vesicles in Immune System Regulation and Type 1 Diabetes: Cell-to-Cell Communication Mediators, Disease Biomarkers, and Promising Therapeutic Tools

**DOI:** 10.3389/fimmu.2021.682948

**Published:** 2021-06-09

**Authors:** Giuseppina Emanuela Grieco, Daniela Fignani, Caterina Formichi, Laura Nigi, Giada Licata, Carla Maccora, Noemi Brusco, Guido Sebastiani, Francesco Dotta

**Affiliations:** ^1^ Diabetes Unit, Department of Medicine, Surgery and Neurosciences, University of Siena, Siena, Italy; ^2^ Fondazione Umberto Di Mario, c/o Toscana Life Sciences, Siena, Italy; ^3^ UOC Diabetologia, Azienda Ospedaliera Universitaria Senese, Siena, Italy; ^4^ Tuscany Centre for Precision Medicine (CReMeP), Siena, Italy

**Keywords:** extracellular vesicles, exosomes, type 1 diabetes, autoimmunity, immune regulation, pancreatic islets

## Abstract

Extracellular vesicles (EVs) are generated by cells of origin through complex molecular mechanisms and released into extracellular environment. Hence, the presence of EVs has been described in multiple biological fluids and in most cases their molecular cargo, which includes non-coding RNAs (ncRNA), messenger RNAs (mRNA), and proteins, has been reported to modulate distinct biological processes. EVs release and their molecular cargo have been demonstrated to be altered in multiple diseases, including autoimmune diseases. Notably, numerous evidence showed a relevant crosstalk between immune system and interacting cells through specific EVs release. The crosstalk between insulin-producing pancreatic β cells and immune system through EVs bidirectional trafficking has yet started to be deciphered, thus uncovering an intricate communication network underlying type 1 diabetes (T1D) pathogenesis. EVs can also be found in blood plasma or serum. Indeed, the assessment of circulating EVs cargo has been shown as a promising advance in the detection of reliable biomarkers of disease progression. Of note, multiple studies showed several specific cargo alterations of EVs collected from plasma/serum of subjects affected by autoimmune diseases, including T1D subjects. In this review, we discuss the recent literature reporting evidence of EVs role in autoimmune diseases, specifically focusing on the bidirectional crosstalk between pancreatic β cells and immune system in T1D and highlight the relevant promising role of circulating EVs as disease biomarkers.

## Introduction

Type 1 diabetes (T1D) is an autoimmune disease characterized by chronic hyperglycaemia, caused by β cells destruction due to specific autoreactive T cells and other immune cells. Despite this simplified definition, the striking heterogeneity of T1D pathogenetic mechanisms has been investigated and reported in multiple studies (1, 2). Such heterogeneity is clinically characterized by different age at onset, disease progression timing and severity (3). The elucidation of additional pathogenetic mechanisms may help to further understand the pathological bases of T1D heterogeneity both at molecular and clinical level.

Extracellular vesicles (EVs) have been recently reported to be involved in multiple diseases and shown to represent pivotal mediators of several pathogenetic mechanisms. Different types of EVs can be identified in biofluids based on their size, content, and surface markers. They are mainly classified in exosomes and ectosomes, both potentially involved in cell-to-cell communication. As a matter of fact, recent evidence demonstrated that under certain conditions, immune cells can communicate with each other and with other cell types through specific mechanisms driven by EVs release and uptake. Specifically, pioneering studies showed that during T1D progression, pancreatic islet infiltrating lymphocytes secrete a distinct subset of EVs capable of transferring a specific cluster of microRNAs (miRNAs) to β cells, thus causing apoptotic pathways activation. On the other hand, β cell EVs release may influence immune cells function through direct lymphocytes activation, or distinct EVs containing autoantigens release and subsequent uptake by local antigen-presenting cells (APCs). Taken together, such studies indicate that an active communication channel between β cell and immune system, based on EVs secretion, may be involved in the pathogenesis and progression of T1D. Of note, it has been also demonstrated that EVs released by pancreatic β cells can contain autoantigens which are transported to neighboring immune cells, thus potentially contributing to the typical autoimmune response (4–6).

Considering EVs ability to mediate signals exchange among different cell types of multiple tissues/organs, it is conceivable that EVs cargo could be an interesting tool to evaluate pathogenetic mechanisms involved in T1D and other autoimmune diseases as well as for the identification of potential biomarkers for the prediction and the progression of the disease. The communication features of EVs could also be used to develop novel therapeutic tools thus opening the way to innovative avenues for the cure or prevention of T1D. As a matter of fact, several evidence of EVs therapeutic ability to induce immunomodulatory effects or immunotolerance have been reported in different diseases.

## Extracellular Vesicles (EVs) Biogenesis, Classification, and Composition

Extracellular vesicles (EVs) are a highly heterogeneous population of phospholipid bilayer membrane-enclosed vesicles, currently classified based on their biogenesis, release mechanism, size, content, and function (7–10). Although the classification of EVs is continuously evolving, they are usually classified into two major categories: ectosomes and exosomes (11, 12).

Ectosomes are directly shed from the plasma membrane and include microvesicles and large vesicles ranging from ~50 nm to 1 μm in diameter. On the contrary, exosomes have endosomal origin and are characterized by a ~40 to 160 nm diameter (~100 nm on average) (13, 14). Their biogenesis and secretion include specific intracellular processes determining selective molecules cargo loading that potentially defines their composition and, consequently, their function (15–17). Exosomes are generated through a process of double invagination of the plasma membrane. The first step leading to the invagination process results into *de novo* formation of an early-sorting endosome (ESE) characterized by cell-surface proteins and soluble proteins (18, 19). Moreover, since ESE continuously exchanges cargo constituents with endoplasmic reticulum (ER), trans-Golgi network (TGN), and mitochondria, it is specifically enriched for proteins and molecules typical of these organelles. Subsequently, the early-sorting endosome can mature into a late-sorting endosome through a second invagination of the plasma membrane leading to the generation of the Multi Vesicular Bodies (MVBs) composed by multiple intraluminal vesicles (ILVs). Based on the invagination volume, ILVs show different sizes and content (20, 21).

**Figure 1:**
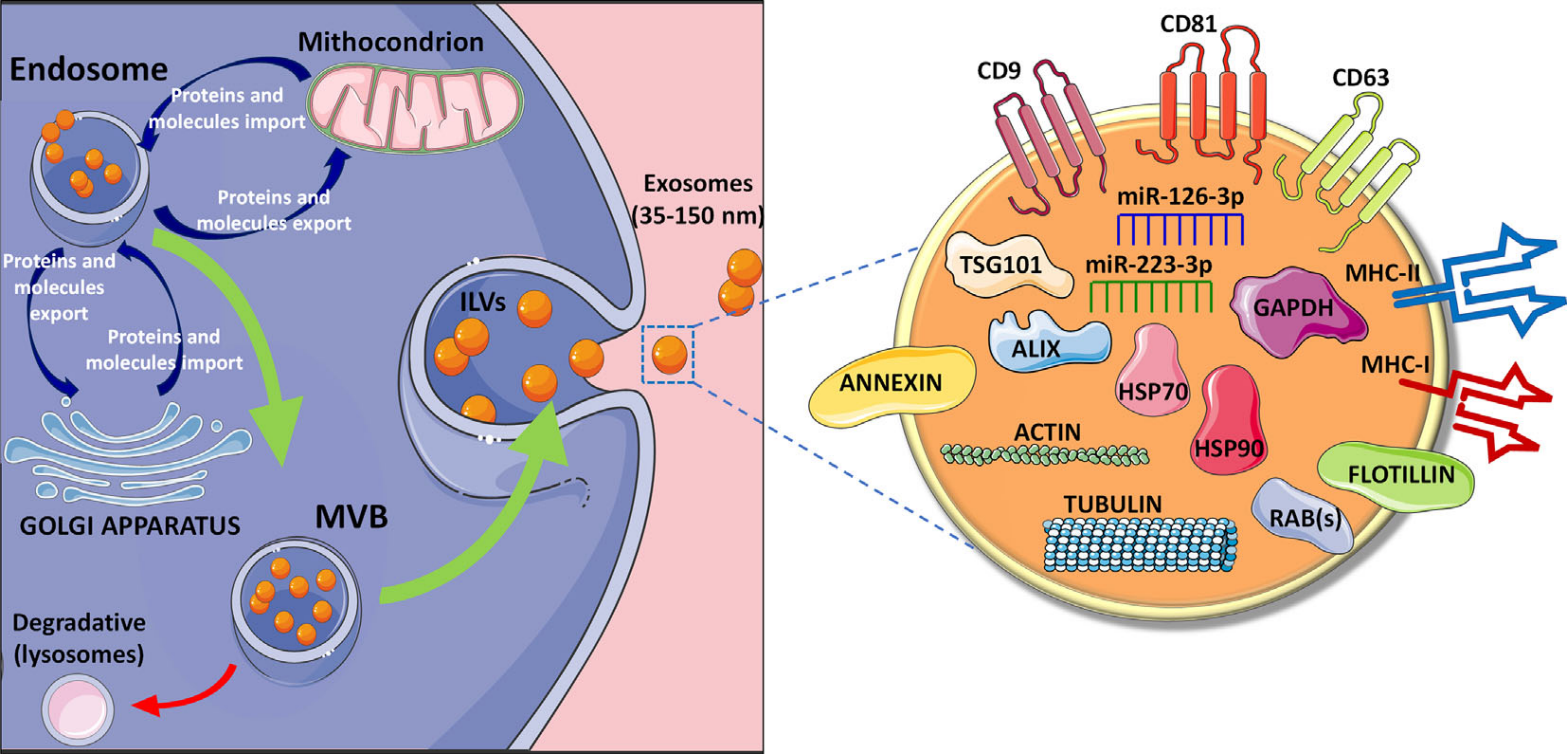
Exosomes biogenesis and composition. Exosomes biogenesis is based on a process of double invagination of the plasma membrane. The first step leading to the invagination process results into *de novo* formation of an early-sorting endosome characterized by cell-surface proteins and soluble proteins. Following the continuous exchange (import and export) of specific proteins and molecules from endosomes and mitochondria as well as with Trans Golgi network, the early-sorting endosome can mature into a late-sorting endosome through a second invagination of the plasma membrane leading to the generation of the Multi Vesicular Bodies (MVBs) composed by multiple intraluminal vesicles (ILVs). Then, MVBs can: (*i*) fuse with lysosomes resulting in the digestion of their ILVs or (*ii*) fuse with the plasma membrane thus leading to the release of ILVs as exosomes (22, 23). Exosomes expose several membrane proteins, such as tetraspanins (CD9, CD81, CD63) (24–28) and MHC molecules, both class I and class II, as well as Annexin and Flotillin. Moreover, exosomes content is highly variable depending on the cell of origin, but usually they contain cytoskeleton proteins (e.g., actin and tubulin), heat shock proteins such as HSP70 and HSP90, proteins associated to metabolism (GAPDH) (29–31), as well as specific proteins (e.g., ALIX, TSG101 and RAB proteins). Exosomes also contains different RNA species such as microRNAs, among which the most reported are miR-126-3p and miR-223-3p (32–36).

MVBs play an essential role both as intermediate progenitor extracellular vesicles and as part of the degradative lysosomal pathway. In details, MVBs can: (*i*) fuse with lysosomes resulting in the digestion of their ILVs or (*ii*) fuse with the plasma membrane thus leading to the release of ILVs as exosomes (**Figure 1**) (22, 23).

The endosomal sorting complex required for transport (ESCRT) plays an essential role in the generation of MVBs. ESCRT mechanism consists of 4 protein subcomplexes (ESCRT-I to IV) acting in a progressive fashion to mediate exosome generation (37–43). The presence of relevant ESCRT proteins in purified exosomes [e.g., tumor susceptibility gene 101 protein (TSG101) and apoptosis-linked gene 2-interacting protein X (ALIX)], represents the evidence of their MVBs origin (18, 44–46). Several studies suggested that ESCRT-independent mechanisms are also involved in MVBs and ILVs biogenesis. These mechanisms involve lipids, tetraspanins and/or heat shock proteins (HSPs) (47–51), thus opening to the presence of multiple parallel mechanisms of exosomes biogenesis.

As a result of their endosomal origin, exosomes carry proteins associated with MVBs formation (Alix and TSG101, as previously cited) and/or in membrane transport and fusion (Rab proteins and annexins) as well as proteins mostly associated with lipid microdomains and exposed to the external exosomes surface, such as integrins and tetraspanins (e.g., CD63, CD9, and CD81) (24–28) (**Figure 1**). However, the identification of novel exosomal molecules is rapidly evolving. Indeed, recent studies demonstrated that Annexin A1 is a specific marker of microvesicles shed from the plasma membrane, while the tetraspanin CD63 is a typical marker of those vesicles released through ESCRT-independent mechanisms, thus rendering these markers not specific for a pure exosome population (52–55). Other proteins identified in almost all exosomes, independently of their origin, are Heat Shock Proteins (HSPs) (e.g., HSP90), cytoskeleton proteins (actin and tubulins), metabolic proteins (GADPH), and major histocompatibility complex (MHC) class I and II molecules (29–31).

Importantly, exosomes also contain different species of RNAs that can be transferred to recipient cells (56–62). Among these, microRNAs (miRNAs), small non-coding RNAs approximately 19 to 24 nts in length, have generated a particular interest in the scientific community for their regulatory function. Two putative pathways of miRNAs sorting into exosomes have been suggested: (i) neural sphingomyelinase 2 (nSMase2)-dependent pathway; (ii) sumoylated heterogeneous nuclear ribonucleoprotein (hnRNP)-based sorting. Although additional studies are needed, it was demonstrated that overexpression of nSMase2 causes an increase in the amount of exosomal miRNAs, while its inhibition leads to a reduced number (63, 64). Regarding hnRNP-based sorting, it has been reported that sumoylated hnRNPA2B1 recognizes the GGAG motif in the 3' portion of miRNAs sequences triggering a specific sorting into EVs (65). Such evidence underlines that the 3'-end of the miRNA sequence contains an important sorting signal motives, which strongly contributes to the miRNAs sorting into EVs (66). However, the presence of specific miRNAs sequence motives is not the only leading cue determining its loading into EVs; indeed, loading mechanisms can also be modulated by miRNA-induced silencing complex (miRISC). Of note, the specific knockout of ribonucleoprotein complex Ago2 (main component of RISC complex) induced the reduction of some EVs enriched miRNAs (miR-142-3p, miR-150, and miR-451) from HEK293T cells (67), thus indicating that the presence of a fully functional miRISC protein complex is fundamental for miRNAs sorting through EVs.

Different miRNAs, such as miR-126-3p and miR-223-3p, have been reported to be enriched and secreted through EVs (**Figure 1**), even though additional confirmatory studies are needed to definitely characterize specific EVs miRNAs content (32–36).

Interestingly, in order to regularly update information about EVs miRNAs, several databases across different biofluids and isolation methods have been generated, such as the extracellular vesicles miRNA database (http://bioinfo.life.hust.edu.cn/EVmiRNA), Vesiclepedia (http://microvesicles.org/), ExoCarta (http://exocarta.org/), and miRDB (http://mirdb.org/) (26, 27, 68, 69).

## EVs Role in Immune System Homeostasis and Immune Cells Crosstalk

In order to support cellular homeostasis and provide host defense, immune system has developed direct and indirect communication mechanisms, such as the release of soluble factors or the transfer of information through EVs (70). The latter has been extensively investigated in macrophages, dendritic cells (DCs), B cells and T cells. Indeed, EVs secreted by all these cells are highly interconnected and act in several molecular mechanisms controlling immune system function, mainly through the modulation of initiation, expansion, and maintenance of innate and adaptive immune responses (9, 71, 72).

Several studies have shown that macrophages infected with different intracellular pathogens, such as *Mycobacterium tuberculosis*, Bacillus-Calmette-Guèrin (BCG), *Salmonella*, *Toxoplasma*, or *Leishmania*, can release EVs carrying pathogens-associated molecular patterns (PAMPs). The exposure of naïve macrophages to such EVs leads to an increased production of cytokines *via* Toll-like receptors (TLR) activation, then promoting the exacerbation of the immune response (73, 74). As a matter of fact, in other two studies, it was demonstrated that EVs released by monocytes exposed to interferon alpha (IFN-α), lipopolysaccharide (LPS), or a combination of both, were able to enhance the production of proinflammatory cytokines by not-treated monocytes as well as by epithelial cells, mainly through the activation of TLR4/nuclear factor kappa-light-chain-enhancer of activated B (NF-kB) cells pathway (75, 76).

Bacteria and parasites can also release specific EVs [e.g., outer-membrane vesicles (OMV)] carrying specific molecules having immunomodulatory properties, such as LPS and lipoproteins (77, 78). Furthermore, bacterial-derived EVs contribute to the exacerbation of bacterial infections by carrying and releasing elements inducing resistance to antimicrobials and complement system factors, as well as by transferring virulent factors. In fact, these EVs seem to be a vehicle for the diffusion of PAMPs which consequently lead to different events: (*i*) triggering of pattern recognition receptors (PRRs) signalling and inflammasome activation; (*ii*) triggering of stimulator of interferon genes (STING) pathways and (*iii*) activation of host innate immune cells, finally leading to the amplification of tissue damage and inflammation (79–82).

Furthermore, macrophage- and monocyte-derived EVs are also involved in inflammatory processes. Indeed, it was reported upon LPS stimulation, they can transfer chemokines, such as tumor necrosis factor α (TNF-α), C-C motif chemokine ligand 3 (CCL3), C-C Motif Chemokine Ligand 5 (CCL5), granulocyte colony-stimulating factor (G-CSF), interleukin-1 receptor α (IL-1Rα), and C-X-C motif chemokine ligand 2 (CXCL2) that induce the synthesis of additional pro- or anti-inflammatory mediators and act as homing signals for other immune cells (83). These data suggest that EVs secreted by activated macrophages strongly work as signal for immune response by host cells (83).

Interestingly, in inflammatory processes, macrophages-derived EVs are also able to induce the activation of memory CD4^+^ and CD8^+^ T cells. In fact, intranasal administration of exosomes isolated from BCG-infected macrophages of *M. bovis* induces the proliferation of memory CD4^+^ and CD8^+^ T cells (84).

In addition to monocytes/macrophages, also dendritic cells (DCs) have been reported to secrete EVs with essential roles for immune regulation processes. DCs, which are the main antigen-presenting cells (APC), canonically activate T lymphocytes through MHC-peptide complex (85). However, APCs can also indirectly activate lymphocyte, through the release of specific EVs (86, 87) carrying the entire MHC-peptide complex (88). However, it was also demonstrated that T cell stimulation by DCs-EVs is less effective than that by APCs (89, 90). It was hypothesized that this weaker stimulatory ability could be ascribed to a low capacity of EVs to T cell receptor (TCR) cross-linking, possibly due to their small size or to a different membrane composition (91, 92).

Given their function in several immune processes, the potential role of DCs-EVs in autoimmunity was taken into consideration for the presentation of intracellular antigens to immune cells. Importantly, several studies have shown that antigens presented by APCs can undergo intracellular transfer, where they are internalized by EVs and delivered to autoreactive T cells (93–95).

As lymphocytes can be targeted and activated by EVs released by other immune cells, they also release specific EVs involved in the crosstalk mechanisms regulating immune response in different contexts. The first evidence of lymphocyte-released EVs was reported in 1996 by Raposo et al., who demonstrated that human B cell–derived EVs are efficiently able to present MHC class II (MHC-II) peptide complexes (pMHC-II) to CD4 T cells *in vitro* (88). Later, it was demonstrated that the release of EVs by primary B cells, requires pMHC-II ligation with cognate TCR on CD4^+^ T cells (96), CD40/IL-4 signalling, and relies on pathways mediated by NF-kB for T cell–induced B cell proliferation (97). Moreover, Rialland and colleagues showed that IgG-mediated B Cell Receptor (BCR) cross-linking in DOHH2 immortalized B cell line, stimulates EVs release, although to a minor extent with respect to TCR–pMHC-II interaction (98), which is the major determinant for EVs release by B cells. As an example, one of the potential targets of B cell–derived EVs are follicular DCs (FDCs) which are activated upon B cell-EVs exposure. As a matter of fact, B cell EVs are specifically uptaken by FDCs which, in turn, present MHC-II peptides to CD4^+^ T cells, leading to T cell differentiation, expansion or silencing depending on the pathological context (99).

An essential role in immune regulation is also played by T cells-derived EVs containing TCR/CD3 complexes (100) and mainly associated to antiviral immune response. It was recently observed that upon activation by DCs, T cells can release EVs containing genomic and mitochondrial DNA, which are then transferred to DCs, leading to the enhancement of antiviral responses *via* the cGAS (cyclic GMP-AMP Synthase)/STING cytosolic DNA-sensing pathway and subsequently inducing interferon regulatory factor 3 (IRF3)-dependent interferon-regulated genes expression (101). These results suggest a potential feedback mechanism by which T cells enhance the activity of APCs, priming them to a more efficient response to the infections (71). We can speculate that this immunomodulatory mechanism could also occur toward Enteroviruses infection, which is one of the most investigated and described environmental risk factor in the pathogenesis of T1D.

Recent studies demonstrated that EVs subpopulations play different roles in immune cells, thus generating multiple immune responses. For instance, Whalund et al. observed that when DCs are loaded with the whole ovoalbumin (OVA) antigen, exosomes from OVA-pulsed DCs are more efficient in inducing antigen-specific CD8^+^ T-cells activation, and in eliciting antigen-specific IgG production respect to other microvesicles types. In this case, this was probably due to the greater ability of exosomes to carry high levels of the antigen, while in other microvesicles it was barely detectable (102).

Furthermore, similarly to healthy cells, also apoptotic cells have been reported to release extracellular vesicles (termed apoptotic extracellular vesicles, Apo-EVs) (103). The release of these EVs seems to occur during apoptosis and differs from apoptotic bodies formation. Apo-EVs have similar characteristics to those EVs released by healthy cells, in terms of cargo delivery and immune regulation; indeed, this peculiar EVs population mainly acts on inflammation, cancer and autoimmunity (103–105). Of note, Apo-EVs formation has also been proposed as a mechanism to facilitate the transport of autoantigens to APC, thus putatively playing a key role in the initiation of autoimmune reaction *in situ*. As an example, Dieudè and colleagues recently discovered a novel class of vesicles, namely apoptotic exosome-like vesicles, much more active than most known apoptotic bodies and released by endothelial cells following serum-free starvation induced-apoptosis. As a matter of fact, the injection of these class of vesicles into C57Bl/6 mice was able to trigger the production of anti-LG3 autoantibodies (subunit of perlecan), thus enhancing the severity of graft rejection. Importantly, apoptotic exosome-like vesicles are particularly enriched of 20s proteasome subunit, and since LG3 behaves as a proteasome substrate, authors further demonstrated that proteasome activity within apoptotic exosome-like vesicles directly regulates anti-LG3 autoantibody formation and allograft inflammation (106).

Overall, these data suggest that immune cells-derived EVs can exert similar functions as their parental cells, including immune stimulation or inhibition. Indeed, the crosstalk between immune cell populations through EVs physiologically contribute to the maintenance of immune system homeostasis, whose alterations can lead to several disorders, including autoimmune diseases.

## EVs and Autoimmune Diseases

Accumulating evidence reveal that EVs from both immune and non-immune cells could play a key role into the regulation of immunity (107). As reported above, EVs are involved in the establishment, maintenance and modulation of autoimmune processes through different mechanisms (108).

EVs can be secreted by APC and may represent a source of autoantigens leading to the activation of autoreactive T lymphocytes (109–111). In fact, several *in vitro* studies showed the ability of APC EVs to directly or indirectly stimulate T cells in order to establish a “dialogue” required for their activation (89, 108, 112). Alternatively, EVs can interact with APC through multiple independent molecular mechanisms involving TCR complexes-MHC interaction (92), co-stimulatory molecules binding (108), or by direct EVs cargo internalization and processing (7, 92). Due to this variety of mechanisms, EVs can contribute to the pathogenetic molecular mechanisms of different immune-mediated diseases including multiple sclerosis (MS), rheumatoid arthritis (RA) and systemic lupus erythematosus (SLE).

Since EVs are enriched in the cerebrospinal fluid (CSF), they could also play a relevant role in nervous system autoimmune diseases such multiple sclerosis (MS) (93). MS, which represents the most common autoimmune demyelinating disease of the CNS (113), characterized by lesions of the blood-brain barrier (BBB) thus contributing to the trans-endothelial migration of autoreactive T cells into the CNS, inducing chronic neuro-inflammation and demyelination (93, 114, 115). In this context, endothelial cells and platelet-released EVs resulted increased in the peripheral blood of MS patients during relapses phases of the disease, consequently causing enhanced endothelial permeability and increased migration of immune cells (116, 117). In addition, it has been demonstrated that EVs released by infiltrating T cells, containing C-C chemokine ligand 5 (CCL5) and arachidonic acid, are able to enhance immune cells recruitment thus contributing to further BBB disruption (117). Finally, microglia-derived EVs, containing MHC class II molecules, also contribute to restimulate infiltrated lymphocytes thus leading to the spreading of neuronal antigens outside the CNS (115, 118).

Another autoimmune disorder in which EVs have been reported to play a pivotal role is rheumatoid arthritis (RA), a chronic inflammatory autoimmune disease characterized by joint inflammation and destruction associated with systemic symptoms (113). Of interest, synovial fluid-derived EVs carry fibrinogen components and vimentin, thus stimulating autoantibodies production and contributing to the generation of immune complexes (110, 119). Therefore, pathogenetic molecular mechanisms of RA are strongly related to EVs-mediated cell-to-cell communication among immune cells, synoviocytes, endothelial cells, and chondrocytes, through different mechanisms (93, 110, 113, 119). On the other side, EVs derived from synoviocytes released in inflammatory conditions may stimulate adjacent cells to secrete mediators of inflammation, leading to cartilage damage. Furthermore, EVs also contribute to RA pathogenesis by promoting the survival of autoreactive T lymphocytes. In fact, inflamed tissue-resident fibroblasts secrete TNF-α^+^ exosomes which are uptaken by autoreactive T lymphocytes, thus becoming resistant to the activation induced cell death (AICD) (120). Moreover, the enzymatic cargo of EVs can also contribute to RA pathogenesis, leading to the degradation of the cartilage matrix caused by the secretion of proteinase and glycosidase enzymes by synovial fibroblasts (115, 121, 122). On the other side, in the RA synovial fluid an increase of leukocyte derived EVs has also been described (123). In particular, EVs derived from T lymphocytes and macrophages are able to induce the secretion of metalloproteinases, proinflammatory mediators, and proangiogenic chemokines by synovial fibroblasts (121). Finally, it can be also hypothesized a role for platelet-derived EVs in RA angiogenesis occurring in synovia, promoting development and growth of synovial membrane and subsequent cartilage and bone destruction as well as articular remodeling (108, 110). Indeed, synovial fluid of RA patients has been reported to contain proinflammatory immune complexes generated by the association of platelet-derived microvesicles and citrullinated peptides autoantibodies (124). Furthermore, Boilard et al. (125) also demonstrated that platelets-released microvesicles play an active role in RA inflammatory processes being detected exclusively in joints of RA patients but not in those from control individuals. Of note, depletion of platelets in K/BxN mice (rheumatoid arthritis animal model), was able to reduce arthritis severity, mainly through the reduction of IL-1 (125).

The same research group also demonstrated that activated platelets are able to release respiratory-competent mitochondria encapsulated in microparticles or as free organelles. Since mitochondria are a substrate for secreted phospholipase A2 IIA (sPLA2-IIA), the hydrolysis of the mitochondrial membrane by sPLA2-IIA yields inflammatory mediators (i.e., lysophospholipids, fatty acids, and mtDNA) that promote leukocyte activation and RA typical inflammation (126).

A specific role of EVs has also been reported in systemic lupus erythematosus (SLE). SLE is a chronic systemic autoimmune disease characterized by the presence of autoantibodies (anti-double stranded DNA and antinuclear antibodies), resulting in the deposition of immune complexes in several tissues, thus consequently leading to organs damage (93, 119). Of note, the role of EVs in SLE has recently been investigated (108). In fact, it has been shown that EVs released from apoptotic cells carry antigenic nuclear determinants, thus being targeted by anti-DNA and anti-nucleosome antibodies from SLE affected mice and from plasma of patients with SLE (127). Moreover, in SLE patients, circulating proinflammatory EVs, are able to induce TNF-α and IFN-α secretion by peripheral blood mononuclear cells (PBMC) through a TLR-mediated mechanism (115, 128, 129). In addition, SLE-specific EVs cargo, composed of metalloproteinases, tissue factor and CD40, are able to induce VEGF production and chemokines secretion thus exacerbating pathological angiogenesis typical of SLE (130, 131). As an example, it has been reported that in advanced SLE vascular lesions, atherosclerotic plaques can release CD40 ligand^+^ (CD40L)-microvesicles which can further stimulate endothelial cell proliferation and angiogenesis, thus contributing to the transition from stable to unstable plaques and to the worsening of SLE vascular lesions (130).

## EVs and Type 1 Diabetes

As already mentioned, EVs are essential components of cell-to-cell communication, thus being involved in the crosstalk between different cell types. Among these, β cells have been reported to actively communicate *via* EVs with other cells, including immune cells. Of note, several recently published studies specifically revealed a bidirectional crosstalk between β cells and immune cells *via* EVs, both in physiological and pathological conditions (**Table 1**).

**Table 1:** EVs are involved in the crosstalk between β-cells and immune cells.

Vesicle type	EVs isolation methods	Cells of origin	Recipient Cells	EVs Function/Cargo	References
Small microparticles	UC	MIN6	Splenocytes	Trigger secretion of inflammatory molecules	132
EVs	DUC	NOD mouse β cells	Tissue-resident APCs	Insulin	4
Exosomes	DUC	MIN6, INS-1 β cell lines and human and rat islets	APCs	GAD65, proinsulin and IA2	5
EVs	UC	Human islets	N/A	GAD65, ZnT8, Glut2	133
EVs	DUC	Human islets	PBMCs	Trigger proinflammatory immune responses	134
Exosomes	ExoQuick-TC	Human islets	N/A	mRNAs, lncRNAs, miRNAs, piRNAs, snoRNAs and tRNAs	135
Exosomes	UC	MIN6, INS-1 and human islets	MIN6 β cells	miRNAs released following cytokines exposure	136
Exosomes	DUC	NOD mouse β cells	Splenocytes	Trigger secretion of TNF-α *via* miR-29b	137
EVs	DUC+UF+SEC	MIN6	APCs derived from bone marrow	Increased cargo of insulin, proinsulin and β cell autoantigens	138
EVs	UC	Human islets	Human islet endothelial cells	Insulin, C-peptide, GLP1R, VEGFa, eNOS, and miR-27b, miR-126, miR-130, and miR-296	139
Exosomes	UC	T-lymphocytes	Rodent and human pancreatic β cells	Trigger chemokine expression and apoptosis of β cells *via* miR-142-3p/-5p and miR-155	6

Table reporting the studies demonstrating a cross-talk among β cells and other cell types, with a potential role in T1D. In details, column 1 lists described EV types; in second column EV isolation method is reported; third and fourth columns respectively illustrate origin and recipient cell types; in fifth column main cargo molecules and/or EVs played role are described; in the last column belonging reference is reported.

UC, ultracentrifugation; DUC, differential ultracentrifugation; UF, ultrafiltration; SEC, size exclusion chromatography.

### EVs From Pancreatic β Cells to Immune Cells

The first evidence of EVs secretion by pancreatic β cells was reported in 2011 (132). In this study, Sheng H. and colleagues showed that the murine β cell line mouse insulinoma-6 (MIN6) was able to secrete EVs characterized by the typical pattern of exosomal markers, alongside with pancreatic islet β cell autoantigens including GAD65. Of note, the exposure of splenocytes obtained from Non Obese Diabetic mice (NOD 6-8 weeks) to EVs isolated from MIN6 cells, induced the secretion of multiple inflammatory molecules including IL-6, IFN-γ, TNF-α, MCP-1, and IL-10. Of interest, cytokines release was completely abolished when splenocytes were isolated from TLR-innate signalling pathway MyD88-KO NOD mice; similarly, lymphocytes proliferation assay showed a significant reduction of the proliferation rate when MyD88-KO NOD mice splenocytes were exposed to MIN6 EVs respect to wild type splenocytes, thus suggesting that the inflammatory stimuli enclosed within MIN6 EVs may activate TLR signals *via* MyD88-dependent pathways, then favoring lymphocytes proliferation. Splenocytes exposure to MIN6 EVs also generated an increase of MHC class II, CD80 and CD86 costimulatory molecules on APC cells, paralleled by an increase of IFN-γ-secreting T cells. Importantly, authors also demonstrated that the intravenous injection of MIN6 EVs in Non Obese Resistant (NOR) mice induced a significant increase in insulitis. Indeed, the severity of insulitis or lymphocyte infiltration was three-fold higher in the NOR mouse group treated with MIN6 EVs for 7 days in comparison to untreated group, demonstrating a tight crosstalk between MIN6 and immune cells activation in autoimmune diabetes through EVs.

**Figure 2:**
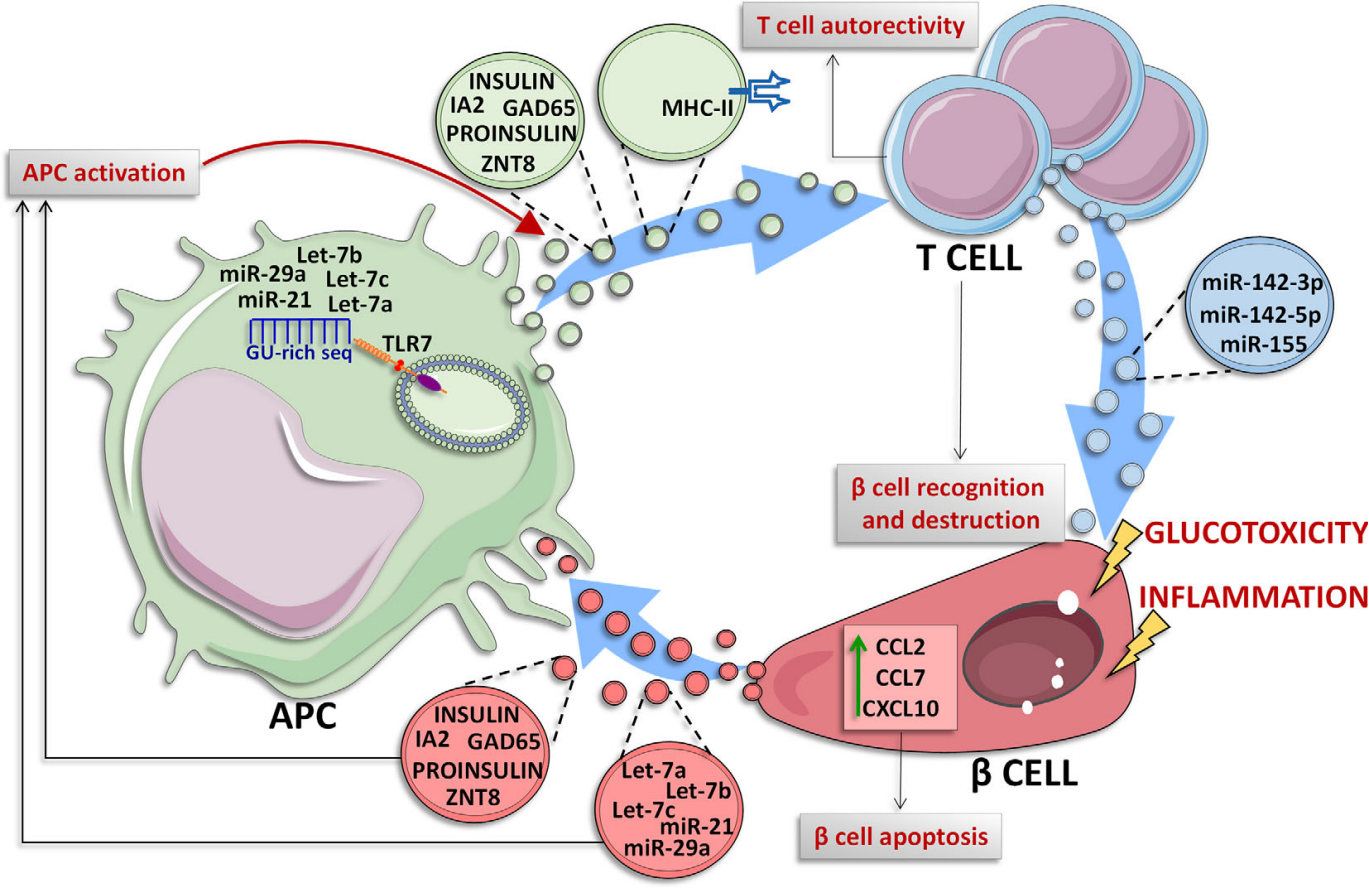
Crosstalk between β cells and immune cells through EVs. In T1D context, β cells and immune cells tightly communicate each other. β cells are subjected to glucotoxic and/or inflammatory stress and can release EVs containing specific miRNAs characterized by a GU-rich sequence (let-7a/b/c, miR-21 and miR-29a) which are transferred to resident Antigen Presenting Cells (APCs), where these miRNAs can bind to endosomal Toll Like Receptor 7 (TLR7) leading to the activation of inflammatory signals (140–142). Moreover, under inflammatory stress, β cells secrete and transfer EVs to APCs. Such EVs can contain specific autoantigens such as insulin, IA2, ZNT8, GAD65 and proinsulin (5) thus leading to their transfer to APC which can present these antigens for adaptive immunity activation. Activated APC can also lead to CD4^+^ T cells activation through two different mechanisms: (*i*) release of EVs containing insulin, IA2, ZNT8, GAD65 and proinsulin autoantigens (4), or (*ii*) release of EVs exposing MHC-II on their surface through which APCs present autoantigens to CD4^+^ T cells (86, 87), leading to autoreactive T cell activation and subsequent β cell destruction. In T1D context, pancreatic islet-infiltrating T cells secrete a specific subpopulation of EVs carrying miR-142-3p/5p and miR-155 which can be transferred to β cells; these miRNAs cause the upregulation of inflammatory molecules such as CCL2, CCL7 and CXCL10 leading to β cell apoptosis (6).

Among EVs cargo molecules released by pancreatic islet β cells, insulin is of particular interest. In 2015, Vomund and colleagues demonstrated that NOD mouse β cells can transfer vesicles containing insulin or its catabolites to tissue-resident APCs. Importantly, this result was confirmed also in human pancreatic islets. In this case, the EVs transfer, which required a close contact interaction between β cells and APCs, was increased upon glucose stimulation and requires mobilization of intracellular Ca^2+^. Additionally, the authors observed that CD4^+^ T cells of diabetic NOD mice exposed to various peptides of the insulin B chain, are able to recognize the APCs vesicles-transferred antigens, thus reporting a potential mechanism through which β cells become recognizable by autoreactive T cells in T1D upon autoantigens transfer through EVs (**Figure 2**) (4).

Recent studies further demonstrated the presence of islet autoantigens in β cell-derived EVs and their specific release upon proinflammatory conditions. Cianciaruso and colleagues showed the presence of islet autoantigens (GAD65, proinsulin, and IA2) in EVs isolated from MIN6 and INS-1 β cell lines supernatant as well as from those isolated from human and rat islets culture medium (5). The induction of ER stress by proinflammatory cytokines in rat islets increased EVs secretion. Of note, mouse islet EVs can be uptaken by APCs leading to their activation. Interestingly, APCs activation was increased when exposed to EVs derived from proinflammatory cytokines treated islets vs. non treated control samples, thus demonstrating that inflammatory stress induced EVs cargo molecules modulation as well.

The presence of GAD65 autoantigens in EVs released by human pancreatic islets was also confirmed by Hasilo and colleagues who were also able to detect the autoantigen ZnT8, as well as the β cell glucose transporter 2 (Glut2) (133).

Human pancreatic islet EVs can be actively internalized by PBMCs, particularly by CD14^+^ cells and by CD19^+^ B-cells. Of note, IL-6, TNF-α and IL-1β cytokines expression were significantly increased in CD14^+^ cells upon EVs internalization. Such inflammatory pathways triggering also involved the upregulation of IFN-γ, IL-4, and IL-17 expression in CD4^+^ T cells and a significant increase of CD19^+^ cell proliferation upon human islets EVs PBMCs exposure. Interestingly, both Th1 cytokines expression and CD19^+^ cells proliferation were further increased in T1D derived PBMCs exposed to human islets EVs. Additionally, human islets EVs are able to specifically activate memory T cells derived from T1D patients but not those from non-diabetic control subjects, as documented by a significant increase of CD69 MFI in both memory T-cells (CD4+CD45RO+CD45RA−CCR7−CD62L−) and B-cells (CD19+IgD−CD27+) observed in T1D PBMCs (134). Since memory B and T-cells activation upon EVs exposure is selective to T1D patients and not observed in non-diabetic controls, these results strongly suggest a pivotal role of EVs in antigen-specific responses.

These data demonstrate the role of EVs in the transfer of autoantigens, or other inflammation-relevant molecules, from islets to immune cells thus representing a novel mechanism of autoimmune reaction triggering in T1D pathogenesis.

As described previously, miRNAs and other RNA molecules are critical components of EVs cargo and could play a key role in the dialogue between pancreatic islets and immune cells (143). Notably, given their intrinsic regulatory function, the evaluation of miRNAs and other small non-coding RNAs enclosed in islet-derived EVs is of particular relevance. In 2019, the group of Evans-Molina characterized the RNA content of exosomes secreted by *in-vitro* cultured human pancreatic islets (135). In this study, Krishnan and colleagues performed both total RNA and small RNA sequencing analysis of human pancreatic islets released exosomes pool, showing a prevalence of mRNA transcript, long non-coding RNAs and miRNAs. Of note, they highlighted the presence of additional small RNA species such as tRNA fragments and piRNAs, showing the high complexity of human islets exosomal RNAome. A critical question related to exosomal RNAome is about its potential alteration following stress stimuli typical of T1D, such as proinflammatory cytokines (IL-1β+ IFNγ). Of interest, they found a total of 133 mRNAs, 31 lncRNAs, 19 miRNAs, 25 piRNAs, 8 snoRNAs, and 20 tRNAs as differentially expressed in EVs secreted from cytokines-treated human pancreatic islets respect to not-treated control samples. Among differentially expressed miRNAs in EVs released from human pancreatic islets in response to cytokines, Krishnan and colleagues identified miR-155-5p as the most upregulated miRNA, while miR-4485 was the most downregulated. Although such data cannot confirm the direct origin of EVs small RNAs content alterations from β-cells, they certainly indicate a global contribution of islet EVs to potential pathogenetic mechanisms and cell-cell communication pathways in T1D. As a matter of fact, other evidence previously showed that exosomal small RNAs cargo is profoundly shaped by proinflammatory stressors in β cells. Importantly, exosomal miRNAs do not simply represent cellular miRNAs expression changes. Guay C. and colleagues demonstrated that MIN6 exosomal miRNAs are significantly different upon proinflammatory stress respect to untreated control and that specific miRNAs are preferentially released in exosomes only after cytokines exposure while others are selectively retained by the cells (136). Of interest, exosomes released from cytokines-treated MIN6 β cells can be horizontally transferred to not stressed MIN6 cells, inducing the same type of apoptotic response and demonstrating also the transfer of exosomes among β cells (136). A point of interest resides in the evaluation of the contribution of small RNAs transfer mediated by exosomes from β cell to other cells. Salama and colleagues demonstrated that the exposure of splenocytes isolated from diabetes-prone NOD mice to β cell-released exosomes is able to increase the synthesis and secretion of TNF-α (137); such effect was dependent on the presence of miR-29b within β cell EVs. The inhibition of exosomal miR-29b reversed the TNF-α increase in splenocytes, thus demonstrating the role of exosomes-transferred miRNAs in the activation of immune cells toward a proinflammatory phenotype.

In the light of the high heterogenous pattern of vesicles secretion by cells of origin, an interesting aspect is the identification of molecules cargo associated to different types of vesicles secreted from the same cell. A recent study analyzed the entire spectrum of vesicle populations (apoptotic bodies, microvesicles and exosomes) secreted by MIN6 cells following the exposure to various stress conditions (proinflammatory stress, UV exposure, hypoxia) (138). Interestingly, the authors reported that proinflammatory stress is able to enhance all EVs population secretion, therefore, leading to an increased vesicles number. Most importantly, EVs released upon inflammatory stress exposure showed changes of cargo molecules respect to the physiologic ones. Of note, apoptotic bodies and exosomes convey not only a higher cargo of insulin, proinsulin, and β cell autoantigens upon stresses but also an increased amount of TLR binding miRNAs (e.g., GU-rich miRNAs let-7a/b/c, miR-21, and miR-29a). TLR binding miRNAs have a specific sequence which can be recognized as a DAMP by endosomal localized TLRs, thus triggering the downstream associated immune signalling. Specifically, Toll-like Receptor 7 (TLR-7) can recognize these EVs-miRNAs thus playing an essential role in the activation of autoimmune processes (140–142). As a matter of fact, EVs secreted from MIN6 cells exposed to inflammatory stress are able to activate primary APCs derived from bone marrow of NOD/ShiLtJ mice (138). Such mechanism is also partially driven by increased cytokines and chemokines cargo levels found in EVs upon MIN6 proinflammatory treatment respect to control. Among them, monocyte chemoattract protein-1 (MCP-1) is one of the most hyperexpressed (138).

Interestingly, it should be noted that human pancreatic islets communicate *via* EVs also with other non-immune cell types. Indeed, Figliolini and colleagues showed that pancreatic islets secreted EVs can be uptaken by local endothelial cells mediating the transfer of several mRNAs (e.g., VEGF and NOS) and miRNAs (e.g., miR-126a, miR-27a), thereby modulating endothelial cells proliferation and function (139). Since human islet endothelial cells represent a pivotal interface directly interacting with immune-cells (144) and playing a critical role in the early phase of T1D by increasing the expression of surface leucocyte-homing receptors, we can speculate that the alteration of pancreatic islet EVs content in response to proinflammatory stressors may indirectly induce endothelial cell dysfunction and, in turn, putative deregulation of their interaction with immune cells. Overall, these studies demonstrated that the specific cargo of different populations of EVs secreted by β cells can activate several immune cell types, thus rendering insulin-producing cells visible and exposed to immune-mediated destruction and leading to the exacerbation of inflammation and autoimmune diabetes.

### EVs From Immune Cells to Pancreatic β Cells

Immune cells are able to secrete unique EVs populations which can be transferred to other cell types, including insulin-producing β cells. A seminal study by Guay and colleagues, reported that three miRNAs, namely miR-142-3p, miR-142-5p, and miR-155, are particularly enriched in T lymphocytes of 8 weeks NOD mice with respect to mouse pancreatic islets (145). Importantly, authors also demonstrated that NOD mice CD4^+^/CD25^−^ T lymphocytes specifically release a population of exosomes enriched of miR-142-3p, miR-142-5p, and miR-155 (6), and that this release is independent from cytokines secreted by infiltrating immune cells, thus hypothesizing that T cells could be able to transfer a specific population of EVs to β cells. In order to test this hypothesis, dispersed murine islets were treated with exosomes isolated from T cells of NOD mice (exoNOD), and MIN6 cells were subjected to EVs isolated from Jurkat human T cell line (exoT). In both cases they observed an increased expression of miR-142-3p/-5p, and miR-155, thus confirming that these miRNAs are actively transferred from T lymphocytes to β cells in autoimmune diabetes. Importantly, a higher apoptotic rate was observed in both dispersed mouse and human islet cells treated with exosomes secreted by activated primary human T cells (exo-hT), thus indicating a detrimental role for this set of miRNA in β cells.

The pro-apoptotic effect of exosomal miR-142-3p/5p and miR-155 is potentially due to their ability to induce the upregulation of several cytokines (CCL2, CCL7, and CXCL10) in murine and human pancreatic islets β cells. As a matter of fact, the *in vitro* inhibition of these three miRNAs was able to reduce apoptosis rate induced by exoT in primary rat islet cells. The same protective effect was also observed in 4-week-old NOD mice upon inhibition of miR-142-3p/5p and miR-155-5p through adenoviral associated miRNA sponges treatment. Of note, the injection of miRNA sponges reduced autoimmune diabetes development rate (10% in AAV-treated mice vs. 60% of not treated mice) *via* a significant decrease of inflammation and insulitis primarily driven by a specific reduction of CXCL10 protein levels in pancreatic islet β cells.

### EVs as a Potential Source of Novel Biomarkers in T1D

EVs’ secretion and their presence in many biological fluids prompted the scientific community to investigate their role as novel biomarkers (146). Indeed, circulating EVs number and content could be altered in several disease conditions, thus representing not only a vehicle of communication and interaction between different cell types but also a possible source of biomarkers (147).

It is well established that the underlying autoimmune process of T1D occurs long before the clinical onset. Efficient biomarkers for the early identification and stratification of high-risk T1D subjects are still lacking (148). Of note, numerous studies explored the potential usefulness of whole plasma/serum miRNAs analysis to detect novel biomarkers of T1D (149–152). However, the detection of miRNAs associated to different plasma/serum components could be the key to uncover novel and more specific biomarkers. In such context, EVs may represent an optimal source of novel and specific biomarkers. As a matter of fact, different studies demonstrated that EVs’ size, number, and enclosed cargo are altered in several diseases, including T1D (153).

In an effort to characterize circulating EVs of T1D patients, Garcia-Contreras and colleagues analyzed EVs isolated using ultracentrifugation approach from plasma of long-standing T1D patients (~25 years disease duration) and age- and gender-matched non diabetic subjects. They observed reduced EVs concentration and increased average size in plasma of T1D respect to CTR subjects (148). Moreover, the same authors found an altered expression of several miRNAs in circulating EVs isolated from T1D subjects. In details, miR-16-5p, miR-302d-3p, miR-378e, miR-570-3p, miR-574-5p, and miR-579 were downregulated, while miR-25-3p was upregulated in EVs from plasma of T1D subjects respect to controls.

In a more recent work, Tesovnik et al. investigated the potential alterations of plasma EVs miRNAs cargo in children with newly diagnosed T1D (<6 months) (nT1D) and in T1D young adults subjects (max 10 years of disease duration, 10y-T1D) in comparison to healthy subjects. Following EVs small RNAs sequencing, the authors found a higher expression of miR-122-5p and miR-192-5p in nT1D cohort compared to controls, as well as the differential expression of several miRNAs in nT1D versus 10y-T1D, namely the downregulation of miR-193b-5p, miR-195-3p, miR-122-5p, and miR-445-5p and the upregulation of miR-21-5p. Furthermore, miR-195-3p and miR-455-5p were both downregulated while miR-185-5p expression was upregulated in 10y-T1D vs control subjects (154).

The importance to distinguish miRNAs enriched in EVs fraction respect to whole biofluid (serum or plasma) was mainly highlighted in a work published by Lakhter and colleagues (155). In details, they reported a significant upregulation of miR-21-5p in EVs secreted by MIN6 murine β cell line, EndoC-βH1 and human pancreatic islets treated with a proinflammatory cytokines mix. Of note, miR-21-5p was increased in whole serum as well as in serum-EVs from NOD diabetic mice respect to control mice. In T1D subjects, the expression of miR-21-5p was discordant between whole serum and serum-isolated EVs; indeed, miR-21-5p resulted significantly reduced in whole serum T1D respect to controls while it was upregulated in serum-isolated EVs, thus demonstrating that analyzing miRNAs expression by distinguishing different fractions (EVs, ribonucleoproteins, lipoproteins, etc.) could be of critical importance in order to identify potential circulating biomarkers of several diseases, including T1D (155).

Another important work published in 2019 by Mirza and colleagues, showed that several miRNAs can be differentially expressed in the EVs derived from breast milk of T1D mothers respect to non diabetic ones. Particularly, they detected a significant hyperexpression of four miRNAs, namely miR-4497, miR-3178, miR-1246, and miR-133a-3p. Importantly, *in vitro* overexpression of miR-4497 and miR-3178 in macrophages resulted in their activation, thus consequently stimulating the secretion of cytokines (TNF-α and IL-1β) (156). Importantly, since it has been previously demonstrated that newborn food intake could affect the potential future development of T1D (157, 158), it could be useful to further investigate the potential role of these breast milk EVs miRNAs in children breastfed by T1D mothers.

Overall, these data demonstrate that the altered expression of miRNAs in EVs of T1D subjects, both recent onset and long standing, could represent valid biomarkers for the prediction and the diagnosis of T1D. However, further studies are needed to better characterize EVs obtained from T1D patients in order to use them as biomarkers.

## EVs as Innovative Therapeutic Tools for Immune System Modulation and T1D

EVs have recently emerged as novel and attractive therapeutic tools in immune therapy, regenerative medicine and drug delivery (159). A growing number of preclinical and clinical studies investigated EVs therapeutic applications in a wide range of diseases including T1D (**Table 2**) (146).

**Table 2:** EVs and therapeutic applications in T1D or in T1D complications.

Cells of Origin	EVs cargo	Target organ	Therapeutic function	References
Human urine-derived stem cells	Growth factor, transforming growth factor-β1, angiogenin and bone morphogenetic protein-7	Kidney	Reduction of the urine volume and urinary microalbumin excretion; prevention of podocyte and tubular epithelial cell apoptosis in diabetic rats.	160
Bone-marrow stromal cells (BMSCs)	miR-145	Brain	Improved functional outcome and promoted neurorestorative effects	161
Human umbilical cord blood-derived EPCs	N/A	Skin	Enhancement of Angiogenesis Through Erk1/2 Signaling	162
Human umbilical cord blood-derived EPCs	N/A	Skin	Enhancement of the proliferation, migration and tube formation of vascular endothelial cells	163
Bone-marrow mesenchymal stem cells	N/A	Brain	Improvement of cognitive impairments by repairing damage neurons and astrocytes	164
HSP20 overexpressing cardiomyocytes	HSP-20	Heart	Improvement of cardiac function and angiogenesis	165
Mesenchymal stem cells	let-7c	Kidney	Attenuation of renal fibrosis	166
Human circulate fibrocyte	HSP90-Alpha; miR-126; miR-130a; miR-132; miR-124a; miR-125b: miR-21	Skin	Activation of diabetic dermal fibroblast; induction of migration and proliferation of diabetic keratinocyte; accelerate wound closure	167
Mouse serum	miR-106b-5p and miR-222-3p	Endocrine pancreas	Improvement of hyperglycaemia *via* pancreatic beta cell proliferation	168
Human bone marrow mesenchymal stem cells	N/A	Endocrine pancreas	Inhibition of immune rejection	169
Endothelial progenitor cells	miR-126 and miR-196	Endocrine pancreas	Enhancement of neo-angiogenesis of human pancreatic islets	170
Adipose tissue‐derived mesenchymal stem cells	N/A	Spleen	Increase of regulatory T‐cell population and their products without a change in the proliferation index of lymphocyte	171

Table reporting the potential therapeutic applications of EVs. In particular, we included: (i) Origin cells or biofluid from which vesicles are isolated; (ii) the main cargo of these vesicles; (iii) organ targeted by the specific EVs population; (iv) potential therapeutic function and/or mechanisms acted by released EVs.

The most investigated source of EVs as therapeutic tools aimed at immune modulation are mesenchymal stem cells (MSCs) (172). As a matter of fact, MSCs are extensively studied for their regenerative and anti-inflammatory roles (173). The therapeutic properties of MSCs have been largely attributed to their differentiation and self-renewal ability, upon specific environmental stimuli, as well as to their immunomodulatory actions, both toward innate and adaptive immunity (172, 174, 175). However, increasing evidence showed that EVs are responsible of most of the beneficial effects of MSCs-based therapy, as demonstrated by the appearance of regenerative effects following administration of MSCs supernatant in damaged tissues (174–176). EVs efficiently mimic the biological activity of parental MSCs and exhibit the same surface receptors, signaling and cell adhesion molecules or associated antigens of parental cells thus representing an interesting cell-free alternative to MSC-based therapy (172, 174). The use of MSC-EVs therapy, although investigated in a small number of clinical trials, showed several advantages over MSC-based therapy, such as higher safety profile, lower immunogenicity, better biodistribution, no need for engraftment, ability to evade from clearance by reticulo-endothelial system, reduced systemic and off-targets side effects, and the capacity to cross biological barriers, while avoiding risks of oncogenesis, embolism, and immune rejection (172, 173, 176–178). Indeed, several promising results in preclinical studies have kicked off numerous clinical trials on the therapeutic use of EVs in several diseases from autoimmune and inflammatory diseases to cancer.

MSC-EVs are reported to induce an imbalance in the secretory pattern of cytokines from immune cells, in favor of anti-inflammatory ones, while inhibiting the secretion of proinflammatory cytokines (172, 174). Of note, MSC-EVs effects on dendritic cells (DCs) seems to be largely mediated by miRNAs (i.e. miR-21-5p, miR-142-3p, miR-223-3p, and miR-126-3p), resulting in the inhibition of maturation, and consequently to an impairment of antigen uptake and antigen presentation capacity (179). Moreover, MSC-EVs stimulate macrophages M2 polarization meanwhile reducing recruitment, activation, and M1 polarization of macrophages and monocytes (180, 181). Furthermore, MSC-EVs inhibit the activation and proliferation of T and B lymphocytes (182), increase the ratio of Treg cells on effector T cells and enhance the function of Treg (171), inhibit maturation of B cells and the consequent immunoglobulin secretion. In addition, MSC-EVs is also able to re-induce self-tolerance through the transfer of anti-inflammatory and tolerogenic molecules into autoreactive T cells, promoting generation of Tregs and apoptosis of activated T cells (182). Taken together, these findings suggest that MSC-EVs directly influence the immune state of adaptive immune cells and promote an anti-inflammatory phenotype of antigen presenting cells (APCs) also inducing immunotolerance of T and B lymphocytes.

Although the use of MSC-EVs in autoimmune diseases therapy is still at early stages, several clinical trials are evaluating their efficacy and safety. As a matter of fact, a first study published by Rahman and colleagues in 2014 demonstrated the immunostimulatory effect of EVs released by mesenchymal stem cells derived from *in vitro* culture of 8-week-old NOD mice pancreatic islets. Indeed, authors demonstrated that vesicles released by these MSCs and injected into 1 week old NOD mice are able to enhance inflammatory reactions, resulting in the increase of cytokines release (IL-6, IFN-γ, TNF-α, and MCP-1), stimulation of surface TLRs and other innate receptors as well as increased B cells proliferation rate. Therefore, authors hypothesized that MSC-EVs could potentially contain and transport immunostimulatory autoantigens, leading to enhanced immune response (183).

However, more recent evidence suggest therapeutic efficacy of EVs in T1D, through immunomodulatory effects (171), protection of pancreatic islets from autoimmune targeting, and consequent slowdown of disease progression (160, 161, 163–167). Furthermore, adipose tissue MSCs-derived EVs were proved to reduce hyperglycaemia and islet degeneration in mice with autoimmune diabetes (168); in addition, the levels of IL-17 and TNF-α were significantly decreased while IL-4, IL-10, and TGF-β levels, as well as Treg number are significantly increased in spleen of EV-treated mice (171).

Of note, EVs could represent a promising tool to overcome possible failure of islet transplantation due to the immune rejection and loss of function of transplanted islets (169). Indeed, MSC-EVs administration alongside with islet transplantation, is able to improve islet functions simultaneously inducing transplant tolerance by enhancing Treg function (184). In accordance with these observations, MSC-EVs treatment resulted in increased graft survival and normalized blood glucose levels in T1D mice (178).

More recently, EVs have also emerged as vectors of bioactive molecules, given their intrinsic stability, biocompatibility, and target homing ability. Several studies investigated whether EVs could deliver anti-inflammatory compounds in central nervous system (185, 186). Indeed, purified EVs can be loaded with therapeutically active cargo molecules *ex vivo*, e.g., through electroporation of small molecules into EVs or, alternatively, the molecules of interest can be incorporated in EVs *in vivo*, during vesicles biogenesis (187).

Despite these encouraging evidence, clinical translation of EVs has been hindered by challenges in isolation, purification, and high-yield production. Dose-finding is also critical for clinical application, since EVs effects seemed to be dose-dependent (188). Moreover, despite quality control and manufacturing practice improvement, EVs heterogeneity is still difficult to eliminate. With the aim to bypass these limitations, researchers have focused on synthetic EVs production, such as liposomes—the most biocompatible and least toxic artificial vesicles created from cholesterol and phospholipids and cholesterol—and biomimetic nanoparticles—which are hybrid nanostructures coated with exosomes-like membranes with desired targeting or functional characteristics (189). Exosome-mimetic nanosystems showed important advantages in production efficiency and functionality (190). Another critical aspect in the clinical use of EVs relates to the integrity of the cargo, which could be reduced/damaged by common loading methods, thus making it crucial to develop novel loading efficiency evaluation systems (191, 192).

## Concluding Remarks

EVs are involved in multiple mechanisms of immune system function and regulation through the transfer of bioactive cargo molecules which include cytokines, chemokines, lipids, metabolites, and small RNAs. The alterations of EVs mediated cell-cell communication mechanisms have been shown to be critical in multiple autoimmune diseases including T1D. Importantly, EVs have been shown to be involved in the bidirectional crosstalk between pancreatic β cells and immune cells, thus representing an interesting mechanism to be further studied during the pathogenesis of T1D.

EVs have been also explored as a novel potential source of disease biomarkers. Indeed, multiple small RNAs have been found altered exclusively in circulating exosomal component; such results indicate the needs to differently fractionate plasma/serum components [EVs, ribonucleoproteins (e.g., AGOs family), lipoprotein particles], in order to discover novel disease biomarkers, whose alteration can be hindered by a dilution effect when analyzing samples as a whole. It should be underlined the urgent need for standardization of isolation and purification of circulating EVs, quality control checks, and storage protocols, as well as the development of sensitive and scalable downstream methodologies to analyze EVs content, including small RNAs, proteins, and metabolites. Moreover, additional studies about the role and functionality of EVs cargo, and in particular on the ability of non-coding RNAs to further regulate cellular processes into recipient cells, are needed in order to shed light onto their mechanisms and functions.

Multiple critical questions surrounding EVs and T1D research (see **Table 3**) are still requiring adequate studies in order to move forward to large-scale clinical application of EVs, as well as to define rigorous criteria for quality control and standardized strategies for quantification and characterization of EVs. Nonetheless, EVs represent a relatively novel and still largely unexplored research field encompassing biomarkers, pathogenetic and therapeutic area in T1D research, thus strongly requiring focused attention by the research community.

**Table 3:** Outstanding questions on T1D and EVs: how to push the field forward?

Key Question	Comments	References
Which is the best methodology to isolate a pure fraction of EVs from cell culture supernatant and plasma?	Absolute purification and isolation of EVs from their origin biological fluids, is an unrealistic goal at present. Each method and approach has advantages, as well as disadvantages and each of this influences the amount, type and purity of recovered EVs. It is important to choose based on the downstream applications and scientific questions of planned experiments.	193, 194
Are there markers characterizing exosomes released by human β-cells? If so, can we specifically isolate them from cell culture supernatant or plasma samples?	It has been reported that exosomes derived from β-cells are enriched of a cargo composed by different specific β-cells markers (insulin and proinsulin); nevertheless, no surface exosomal marker specific for β-cells has been identified yet. However, as future goal, a potential approach could be that of performing a proteomic profile on exosomes isolated from culture medium of pure human β-cell in order to identify a specific β-cell membrane marker on exosomes surface.	4, 5
Which set(s) of stress stimuli drives differential EVs release and content secreted from primary human β-cells?	It has been reported that proinflammatory cytokines mix induces an increase of miR-21-5p in exosomes derived from Endocβ-H1 and human islets, without significant differences in term of quantity and size of exosomes, suggesting a selective enrichment of EVs miR-21-5p in response to inflammatory stress. Moreover, Krishnan et al. demonstrated that exosomes released from human pancreatic islets treated with IL-1β and IFNγ showed differential expression of several RNAs species, with respect to untreated human islets. However a wide range of stress test on primary islets are still missing and require further studies.	135, 155
Can we use engineered EVs to target β-cells or specific immune cell subsets in order to protect β-cells or to restore immune tolerance in T1D?	EVs have also emerged as vectors of bioactive molecules, given their intrinsic stability, biocompatibility and target homing ability. Cantaluppi et al. demonstrated that EVs released by endothelial progenitor cells carried the proangiogenic miR-126 and miR-296 that enhance neoangiogenesis of human pancreatic islets thus improving their function. A potential mechanism to improve β-cell function may reside in the engineering of endothelial progenitor-like EVs overexpressing miR-126 and miR-296 in order to ameliorate islet function.	170

## Author Contributions

GEG, DF, CF, LN, CM, GL, and NB: writing and drafting the manuscript. GS and FD: manuscript writing, drafting and revising. All authors contributed to the article and approved the submitted version.

## Funding

The work is supported by the Innovative Medicines Initiative 2 (IMI2) Joint Undertaking under grant agreement no. 115797-INNODIA and no. 945268 INNODIA HARVEST. This joint undertaking receives support from the Union’s Horizon 2020 research and innovation programme and EFPIA, JDRF, and the Leona M. and Harry B. Helmsley Charitable Trust. This work is also supported by PON “Ricerca e Innovazione” 2014-2020 (ARS01_01270-IDF SHARID—Innovative Devices For SHAping the RIsk of Diabetes). FD was supported by the Italian Ministry of University and Research (2017KAM2R5_003) and by Italian ministry of Health (PROMETEO). GS was supported by the Italian Ministry of University and Research (201793XZ5A_006) and by Italian Ministry of Health Ricerca Finalizzata 2018” (GR-2018-12365577).

## Conflict of Interest

The authors declare that the research was conducted in the absence of any commercial or financial relationships that could be construed as a potential conflict of interest.
